# Effect of obesity on sleep quality, anthropometric and autonomic parameters in adolescent

**DOI:** 10.5935/1984-0063.20200037

**Published:** 2020

**Authors:** Bruna Cruz Magalhães, Nivaldo de Jesus Silva Soares Júnior, Carlos Alberto Alves Dias Filho, Rafael Martins Andrade, Carlos José Moraes Dias, Silvana de Figueredo Alencar de Oliveira, Luanda Sinthia Oliveira Silva Santana, Carlan da Silva Sena, Janaína de Oliveira Monzani-Brito, Andressa Coelho Ferreira, Cristiano Teixeira Mostarda

**Affiliations:** 1 Federal University of Maranhão, Department of Nutrition - São Luís - Maranhão - Brazil.; 2 Federal University of Maranhão, Physical Education Department - São Luís - Maranhão - Brazil.; 3 Federal University of Maranhão, Laboratory of Cardiovascular Adaptations to Exercise (LACORE) - São Luís - Maranhão - Brazil.

**Keywords:** Sleep, Autonomic Nervous System, Obesity, Adolescent

## Abstract

**Objective:**

To compare the effects of obesity on sleep quality, the anthropometric and autonomic parameters of adolescents.

**Material and Methods:**

A cross-sectional study was carried out with adolescents aged 11 to 18, analyzing parameters such as BMI, sleep quality records, waist circumference, fat percentage, blood pressure and sexual maturation, in addition to autonomic cardiac function through the analysis of heart rate variability.

**Results:**

The anthropometric parameters of waist circumference, percentage fat mass, were signiﬁcantly higher in the group of obese adolescents. Sympathetic modulation in LF% was signiﬁcantly higher in obesity. Parasympathetic modulation in HF% was signiﬁcantly lower in obese than in eutrophic.

**Conclusion:**

Obese adolescents do not have poor sleep quality; there is no distinction between boys and girls regarding the analyzed variables; however, obesity alone was responsible for negatively inﬂuencing anthropometric parameters, as well as impairing the autonomic cardiac modulation.

## INTRODUCTION

Obesity is considered a current pandemic and a serious public health problem. In recent years, the number of obese children has increased am+ong 12 to 17-years-old expressed in 8.4% of the population, 17.1% are overweight, 9.6% are hypertensive^1^. It is known that obesity in adolescents is associated with risk factors for the development of cardiovascular diseases in adult life^2^.

Excess weight when added to increased waist circumference and high fat percentage, increase blood pressure, cardiac output, intravascular volume and sympathetic nerve activity. This picture of autonomic imbalance reduces the cardioprotective action of the parasympathetic nervous system, considered an important risk factor for cardiovascular diseases^3^.

In addition, this clinical state compromises sleep quality and is further aggravated by sleep deprivation and daytime sleepiness. There are also studies that point out poor sleep quality as a preponderant factor for cardiac autonomic dysfunction in this public^4^.

Studies on the effect that obesity causes under autonomic parameters have also been encouraged, and much more in this specific population of children and adolescents. Thus, the most used tool is the analysis of heart rate variability (HRV), which quantifies cardiac sympathetic and parasympathetic modulation^5^.

Studies on the role of obesity in autonomic heart rate modulation in adolescents are of relevance in the public health field, since obesity and autonomic dysfunction are directly correlated to morbidity and early mortality. Also, gaps in the current literature exist related to the autonomic nervous system performance in heart rate modulation among obese adolescents with cardiovascular parameters of elevation of blood pressure, waist circumference and fat percentage. It is also important to note that the autonomic modulation varies with the gender difference^6^, but few studies make this distinction. Thus, this factor must also be considered.

For this reason, the objective of the present study was to compare the effects of obesity on sleep quality, the anthropometric and autonomic parameters of adolescents.

## MATERIALS AND METHODS

This is a cross-sectional study that took place between March and April 2016 in public and private schools of the educational network of São Luís - MA city, in Brazil, with 212 adolescents as a convenience sample. They were divided in three groups: eutrophic, overweight and obesity. The following inclusion criteria was adopted: adolescents of both sexes, aged 11 to 18 years, body mass index (BMI) expressed in Z-score values, using a reference from the WHO^7^, eutrophy ≥ -2 and < 1; overweight Z-score ≥ 1 and < 2; and obesity Z-score ≥ 2. Those with physical disabilities that prevented the measurement and pregnant women were not included.

Recruitment was carried out from 147 schools in the urban and rural areas, from public (municipal, state and federal) and private schools. The sample was stratified by type of school and geographic location. Ten schools were systematically selected, 7 were public and 3 were private, and of these, 8 were located in the urban area. The classes were randomly selected.

All participants, parents and/or guardians were informed in writing through the Free and Informed Consent Form. The protocol of the procedures was approved by the Ethics and Research Committee of the Federal University of Maranhão - UFMA, Brazil.

### Sleep quality

Sleep quality and occurrence of sleep disorders were evaluated using the Pittsburgh Sleep Quality Index (PSQI), as originally defined by Buysse. The PSQI uses seven components: (a) subjective quality of sleep, (b) sleep latency, (c) duration of sleep, (d) habitual sleep efficiency, (e) sleep disorders, (f) use of medication to sleep, and (g) daytime sleepiness and disorders during the day. The score for each component was determined separately, on a scale of 0 to 21 points, where greater the value of the score obtained, worse is the quality of sleep. Score values between 0 and 4 represent good sleep quality, those between 5 and 10 represent poor sleep quality, and those greater than 11 indicate sleep disorders.

### Anthropometric Parameters

#### Body Mass Index

A scale of the brand Filizola^®^ with a capacity of 150kg and a sensitivity of 0.1kg was previously calibrated. To measure body weight, the volunteers had to be free of accessories and wearing light clothes. The stature was verified with a Trena EST 23^®^ compact stadiometer, in the millimeter scale with a capacity of 2.10 meters, coupled to the balance, and the voluntary should be with the eyes fixed in the horizontal plane, barefoot and have the weight equally distributed between the feet, the arms extended along the body and heels together, touching the vertical rod of the stadiometer. With these measures, the body mass index (BMI) was calculated. To classify nutritional status through BMI was obtained from the weight ratio (kg) by the square of the height (m). The BMI classification for adopted age was: eutrophy ≥ -2 and < 1; overweight Z- score ≥ 1 and < 2; and obesity Z-score ≥ 2^7^.

#### Waist circumference

Measurements of waist circumference were made at the border of the iliac crest with inextensible tape measure at the time of expiration.

#### Fat Percentage

The fat percentage was determined with the use of the Maltron^®^ tetrapolar bioimpedance equipment. Such equipment generates a current of 800µA with a frequency of 50kHz. Before this procedure, subjects were instructed to remove all metallic objects and not drink alcoholic beverages and/or caffeine in the previous 24 hours and urinate 30 minutes before the evaluation and did not practice physical activity.

#### Hemodynamic parameters

For blood pressure measurements, two automated blood pressure monitors were used (Omron^®^ HEM-711 and Omron^®^ 905)^8^. The protocol for systolic blood pressure (SBP) and diastolic blood pressure (DBP) measurement followed the norms of the VII Brazilian Hypertension Guideline^9^ and the IV Report on the Diagnosis, Evaluation, and Treatment of Hypertension in Children and Adolescents. An optimal cuff size was used according to the arm size of the participants and the measurement was taken three times^10^.

#### Cardiac Autonomic Parameters

The heart rate variability (HRV) method, which consists of the analysis of different parameters based on the time variation between successive heartbeats, was used to track autonomic cardiac function. It has been used to quantify autonomic cardiac sympathetic and sympathetic cardiac modulation^5^. To track cardiac autonomic function variability was recorded with a 12-lead electrocardiogram of WinCardio^®^ 6.1.1 and the 600Hz electrocardiogram (Micromed Biotechnology Ltd.) signal in the supine position for 10 minutes at rest with frequency spontaneous and normal breathing (between 9 and 22 breaths per minute). The indices were evaluated using Kubios HRV Analysis software version 2.0 (The Biomedical Signal and Medical Imaging Analysis Group, Department of Applied Physics, University of Kuopio, Finland).

Frequency indices obtained by the HRV spectral analysis technique were used in the analysis of the HRV. Stationary periods of the tachogram, with at least 5 minutes, were decomposed in the low and high frequency bands (LF and HF, respectively), which represent the sympathetic and vagal modulations, respectively, and the LF/HF ratio. The bands of interest were: VLF (0-0.04Hz), LF (0.04-0.4Hz) and HF (0.15-0.4Hz)^11^.

#### Sexual maturation

For the evaluation of a sexual maturation, the criteria used by Tanner were adopted. This is a method of self-evaluation using images, which takes into account the development of breasts in girls and that of the penis in boys, as well as hair in both genders.

The development of organs consists of 5 stages: stage 1, represents the pre- pubertal state; stage 2, indicates the beginning of maturational development; stages 3 and 4, indicate continuity not maturational process; and stage 5, adult mature status indicator^12^.

#### Statistical analysis

Statistica^®^ software version 12.0 was used. Data were expressed as mean ± standard deviation. Data were expressed as mean ± standard error. To compare the groups, One-way ANOVA and two-way ANOVA with Tuckey post hoc tests were used to evaluate the difference among groups. The level of significance was set at 5%. To evaluate the association between qualitative variables, the chi-squared test was performed.

#### Ethical aspects

After receiving information about the research objectives, the protocol and the procedures to be carried out, as well as the risks and benefits of participating in the study, the legal heads and directors of the educational institution were handed out printed information sheets and requested to be signed by Free and Informed Consent Form. From the chosen institutions, the authorization of the directors was obtained and adequate forms of approach of the adolescents were established so as not to compromise the progress and routine of the school activities.

Ethical aspects were respected in accordance with CNS Resolution Nº 466 of December 12, 2012 and Resolution CNS Nº 441 of May 12, 2011. The research project was submitted to the Research Ethics Committee of UFMA and approved under protocol Nº-378142.

## RESULTS

From total of 222 adolescents (106 eutrophic, 63 overweight and 53 obese) presented inclusion criteria, only 212 (102 eutrophic, 60 overweight and 50 obese, 57 male and 155 female) participated in the study. During the data collection, 10 subjects were excluded from the study: 4 adolescents became pregnant, 3 migrated to another school, 1 died and 2 gave up evaluations.

Table 1 characterizes the sample expressing the means of the cardiovascular indicators.

**Table 1:** Mean values and standard deviations of anthropometric and hemodynamic indicators of adolescents.

Variables	Mean ± Standard deviation
Age (years)	15.6 ± 1.9
Systolic blood pressure (mmHg)	113.1 ± 12.0
Diastolic blood pressure (mmHg)	66.1 ± 8.0
Heart rate (bpm)	78.2 ± 13.4
Weight (kg)	55.7 ± 10.8
Height (m)	162.9 ± 9.0
Waist circumference (cm)	70.75 ± 7.89
Body fat (%)	20.1 ± 6.1
Z-score	0.07 ± 1.1

Z-score for the BMI parameter.

The results of the comparison of anthropometric and sleep quality parameters of eutrophic, overweight and obese adolescents are shown in Table 2.

**Table 2:** Comparison of anthropometric, sleep quality parameters and autonomic parameters of eutrophic, overweight and obese adolescents.

Variable	Eutrophic (n=102)	Overweight (n=60)	Obese (n=50)	
Age (years)	15.17 ± 0.19	15.96 ± 0.25*	16.06 ± 0.27*	
Weight (kg)	52.7 ± 2.3	59.4 ± 3.1*	61.1 ± 2.3*	
Height (cm)	161.2 ± 4.8	162.3 ± 5.6	161.8 ± 4.3	
WC (cm)	66.68 ± 0.64	72.11 ± 0.83*	77.67 ± 0.91*†	
BF (%)	21.61 ± 0.89	26.45 ± 1.16*	33.14 ± 1.27*†	
Z-score	-0.35 ± 0.75	1.03 ± 0.73*	2.21 ± 0.21*^†^	
PSQI				
Sleep duration	8.17 ± 0.42	9.57 ± 0.74	6.83 ± 0.84	
Sleep efficiency	92.69 ± 1.74	94.82 ± 3.04	87.65 ± 3.78	
Tanner's sexual				
Maturation index				X^2^
1	3 (3.23 %)	4 (2.78%)	3 (3.98 %)	
2	5 (2.59 %)	2 (2.23%)	1 (3.19 %)	[3.95]
3	32 (35.56 %)	30 (30.60%)	48 (43.83%)	
4	3 (1.62%)	1 (1.39%)	1 (1.99%)	

*p<0.05 One-way ANOVA with Tuckey post hoc tests test vs. Eutrophic; †*idem* vs. Overweight. WC: Waist circumference; BF (%): Percentage of fat mass. To evaluate the association between qualitative variables, the chi-squared test was performed; Z-score for the BMI parameter.

Autonomic parameters of eutrophic, overweight and obese adolescents are shown in Table 3.

**Table 3:** Comparison of autonomic parameters of eutrophic, overweight and obese adolescents.

Variable	Eutrophic (n=102)	Overweight (n=60)	Obese (n=50)
SBP (mmHg)	112.07 ± 1.18	113.07 ± 1.54	115.45 ± 1.69
DBP (mmHg)	65.45 ± 0.79	65.45 ± 1.03	67.93 ± 1.13
HR (bpm)	74.77 ± 1.83	75.59 ± 1.44*	81.04 ± 1.41
SDNN (ms)	63.03 ± 3.97	53.20 ± 3.62	54.02 ± 2.78
RMSSD (ms)	63.62 ± 4.26	52.46 ± 3.89	48.32 ± 2.98
LF (ms^2^)	678 ± 79.20	747.10 ± 60.74*	959.18 ± 86.76†*
LF (%)	42.44 ± 1.51	44.98 ± 1.96	47.98 ± 2.15*
HF (ms^2^)	1347 ± 140.99	1154.71 ± 98.71	990.66 ± 128.71
HF (%)	57.55 ± 1.51	55.01 ± 1.96	52.21 ± 2.15*
LF/HF	0.86 ± 0.07	1.06 ± 0.09	1.10 ± 0.10

*p<0.05 One-way ANOVA with Tuckey post hoc tests test vs. Eutrophic; ^†^*idem* vs. Overweight. SBP: Systolic blood pressure; SBD: Diastolic blood pressure; HR: Heart rate; SDNN (ms): Standart Deviation of all normal NN interval; RMSSD (ms): Root Mean of square sucessive NN interval difference; LF: Low frequency; HF: High frequency; LF/HF: Index low frequency and high frequency.

The anthropometric parameters of waist circumference and fat percentage were significantly higher in the group of obese adolescents compared to the overweight and eutrophic adolescents group. The mean of sleep quality indicators did not indicate sleep disturbances in either group.

Both heart rate and sympathetic activity were significantly higher in obese adolescents than eutrophic. And the parasympathetic activity was significantly lower in obese than in eutrophic.

The results of the comparison of sleep quality and anthropometric parameters are shown in Table 4 and not showing statistically significant differences.

**Table 4:** Comparison of sleep quality and anthropometric parameters of eutrophic, overweight and obese boys and girls.

Variables	(Male N=30)	(Female N=72)	(Male N=15)	(Female N=45)	(Male N=12)	(Female N=38)
Age (years)	14.75 ± 0.31	15.41 ± 0.23	15.31 ± 0.40	16.34 ± 0.30	15.14 ± 0.51	16.41 ± 0.31
Height (cm)	161.2±3.23	159.4±2.12	160.4±4.1	162.3±3.6	160.2±2.6	161.5±3.4
Weight (kg)	51.6±4.6	54.4±3.2	59.7±6.8	59.9±7.4	61.5±5.8	62.9±6.3
WC (cm)	67.88 ± 1.06	66.00 ± 0.80	74.28 ± 1.37	70.85 ± 1.04	78.30 ± 1.72	77.43 ± 1.07
BF (%)	16.54 ± 1.40	24.49 ± 1.05	23.40 ± 1.81	28.21 ± 1.38	30.46 ± 2.27	34.18 ± 1.42
Z-score	-0.02 ± 0.25	0.18 ± 0.18	1.46 ± 0.32	1.54 ± 0.24	2.18 ± 0.40	2.59 ± 0.25
PSQI	2.35 ± 0.22	1.70 ± 0.16	2.27 ± 0.28	1.89 ± 0.21	1.42 ± 0.35	1.68 ± 0.22

Waist circumference; BF (%): Percentage of fat mass. *p<0.05 Two-way ANOVA with Tuckey post hoc tests. Z-score for the BMI parameter.

We observed in Table 5, that even with differences between men and women, they were not statistically different in hemodynamic and autonomic parameters.

**Table 5:** Comparison of the autonomic parameters of eutrophic boys and girls, with overweight and obesity.

Variables	(Male N=30)	(Female N=72)	(Male N=15)	(Female N=45)	(Male N=12)	(Female N=38)
Age (years)	14.75 ± 0.31	15.41 ± 0.23	15.31 ± 0.40	16.34 ± 0.30	15.14 ± 0.51	16.41 ± 0.31
Height (cm)	161.2±3.23	159.4±2.12	160.4±4.1	162.3±3.6	160.2±2.6	161.5±3.4
Weight (kg)	51.6±4.6	54.4±3.2	59.7±6.8	59.9±7.4	61.5±5.8	62.9±6.3
WC (cm)	67.88 ± 1.06	66.00 ± 0.80	74.28 ± 1.37	70.85 ± 1.04	78.30 ± 1.72	77.43 ± 1.07
BF (%)	16.54 ± 1.40	24.49 ± 1.05	23.40 ± 1.81	28.21 ± 1.38	30.46 ± 2.27	34.18 ± 1.42
Z-score	-0.02 ± 0.25	0.18 ± 0.18	1.46 ± 0.32	1.54 ± 0.24	2.18 ± 0.40	2.59 ± 0.25
PSQI	2.35 ± 0.22	1.70 ± 0.16	2.27 ± 0.28	1.89 ± 0.21	1.42 ± 0.35	1.68 ± 0.22

SBP: Systolic blood pressure; SBD: Diastolic blood pressure; HR: Heart rate; SDNN (ms): Standard deviation of all normal NN interval; RMSSD (ms): Root mean of square successive NN interval difference; LF: Low frequency; HF: High frequency; LF/HF: Index low frequency and high frequency. *p<0.05 Two-way ANOVA with Tuckey post hoc tests.

## DISCUSSION

The objective of this study was to compare the effects of obesity on sleep quality, anthropometric and autonomic parameters in adolescents. The main finding of this study shows that obese adolescents have impaired autonomic modulation, regardless of sleep quality. Our data shows, sleep quality index values were similar to data from Knutson^13^ and highlight the association between short duration of sleep (usually < 6 hours per night) with increased body mass index or obesity. Experimental sleep deprivation studies demonstrate an autonomic imbalance indicative of decreased parasympathetic activity and/or increased sympathetic activity due to poor sleep quality^13^.

Furthermore, increased weight, waist circumference and fat percentage have been considered a major cardiovascular risk, since they are associated with increased sympathetic nerve activity and reduced parasympathetic activity. The greater waist circumference was related to the lower parasympathetic modulation presented, consequently, a greater autonomic cardiac dysfunction^14^. The increase in weight and percentage of fat can also compromise the modulating function of the autonomic nervous system^15^.

It is worth considering that obese adolescents have sympathovagal imbalance, particularly at night, compared with non-obese adolescents^16^. Although sleep time had a significant effect on the prevalence of obesity^17^, sleeping difficulty has not been frequent among afrodescendants. This may explain why we did not find indices of poor sleep quality in this study, since our sample was made up of afrodescendant adolescents.

Another variable in which obesity did not influence were blood pressure levels. The sympathetic nervous system activity influences hypertension status and systolic blood pressure (SBP) independent of adiposity in youth ranging from normal- weight to severe obesity^18^. Obese youngsters with paternal history of hypertension had significantly higher heart rate, DBP, LF, LF/HF ratio and reduced total power, HF, SDNN, RMSSD compared to the other groups with normal BMI^19^.

In our study, there was no significant difference in relation to blood pressure, but with regard to heart rate, obese adolescents had higher levels than eutrophic and overweight adolescents, with a statistically significant difference in this parameter. It is known that the behavior of the heart rate reflects the sympathetic hyperactivity, as well as the decrease of the parasympathetic activity^16^. This may be justified by the fact that the eutrophic group have a higher parasympathetic modulation, expressed by RMSSD.

In the analysis of HRV in the frequency domain, the sympathetic modulation expressed in LF (ms^2^) and LF (%) was higher in the group of obese adolescents, as well as the reduction of the parasympathetic modulation of HF (%). Obese adolescents have higher LF values, compared to non-obese ones^20^. This happens because obesity in young people is associated with increased sympathetic tone and reduction of vagal tone^21^.

There was no significant difference in sleep quality, anthropometry and in the HRV between eutrophic and overweight boys and girls, even though there is no statistically significant difference HF (ms^2^) was higher in girls than in boys and LF/HF ratio was lower in girls than in boys. The best HRV in girls compared to boys can be explained due to the difference in their hormonal profile. Testosterone increases sympathetic activity and estrogen decreases sympathetic activity^22^. We hypothesize that the lack of significant difference between girls and boys in our study may be due to the optimal levels of sex hormones in this age group. Despite being on stage 3, all the participating girls have informed that menarche has already occurred. This question was made by a woman researcher, avoiding any constrainment.

To better understand the difference of results between the methods used for HRV analysis, it is important to stress that the significant relationship between autonomic modulation and body mass index is not clear. One possible explanation is that body mass index does not have the capacity to precisely quantify body fat, which in turn consists of fat cells responsible for secreting various adipokines, among them leptin, which is responsible for activating the neural pathways, which increase the activity of the sympathetic nervous system^23^.

That is, the distribution of fat and amount of adipose tissue needs to be considered for a better analysis of the influence of obesity over the autonomic parameters, an example of densitometry with dual energy X-ray emission. This indicates another two limitations of this study: double beam absorptiometry was not used to analyze body composition and blood collection was not performed, which would allow the determination of inflammatory markers, hormones and adipokines, allowing the understanding of the mechanisms involved in the results found.

These results warn of the need for actions that seek early action and prevent the evolution to cardiovascular complications. The HRV test may play an important role in the detection of autonomic cardiovascular disorder. However, to be used clinically, other important factors that may influence a HRV such as sex, sleep quality and BMI should be considered. As far as we know, there is a paucity of data that refers to three factors simultaneously in adolescents. Thus, our study appears to be the first to investigate all three factors with a HRV in adolescents.

These results alert to the need for early actions that prevent the evolution to cardiovascular complications, since it is important to increase the components of high frequency (HF) and reduce the components of low frequency (LF) of obese young people, avoiding early impairment of the cardioprotective action of the parasympathetic nervous system in these individuals.

## CONCLUSION

Thus, we conclude that obesity alone was responsible for negatively influencing anthropometric parameters in the study population, as well as impairing the autonomic cardiac modulation of obese adolescents.

### Limitation of the study

The blood pressure of the study participants was not checked at the same time for everyone. Even followed the norms of the VII Brazilian Hypertension Guideline, it can represent a limitation once the cardiovascular parameters have a circadian cycle.

Another limitation can be related to the higher number of female adolescents. However, the intention of the study was to evaluate only the difference between eutrophic, overweight and obese adolescents.
